# Preventative Effects of Antioxidants against PM_10_ on Serum IgE Concentration, Mast Cell Counts, Inflammatory Cytokines, and Keratinocyte Differentiation Markers in DNCB-Induced Atopic Dermatitis Mouse Model

**DOI:** 10.3390/antiox11071334

**Published:** 2022-07-06

**Authors:** Mi Hee Kwack, Jin Seon Bang, Weon Ju Lee

**Affiliations:** 1 BK21 FOUR KNU Convergence Educational Program of Biomedical Sciences for Creative Future Talents, Department of Immunology, School of Medicine, Kyungpook National University, Daegu 41944, Korea; go3004@knu.ac.kr; 2 Department of Dermatology, School of Medicine, Kyungpook National University, Daegu 41944, Korea; jinsun9367@hanmail.net

**Keywords:** antioxidants, 2,4-Dinitrochlorobenzene (DNCB)-induced atopic dermatitis mouse, particulate matter (PM)_10_

## Abstract

Particulate matter (PM) can cause oxidative stress, inflammation, and skin aging. We investigated the effects of antioxidants such as dieckol, punicalagin, epigallocatechin gallate (EGCG), resveratrol, and Siegesbeckiae Herba extract (SHE) against PM < 10 μm (PM_10_) on serum IgE concentration, mast cell counts, inflammatory cytokines, and keratinocyte differentiation markers in a 2,4-Dinitrochlorobenzene (DNCB)-induced atopic dermatitis mouse model. Seven-week-old BALB/c mice were sensitized with 2% DNCB. Atopic dermatitis-like lesions were induced on the mice with 0.2% DNCB. Antioxidants and PM_10_ were applied to the mice for 4 weeks. PM_10_ increased the serum IgE concentration and spleen weight in mice, and all antioxidants downregulated these parameters. Histological examination showed an increase in epidermal thickness and mast cell counts in response to PM_10_, and all antioxidants showed a decrease. PM_10_ upregulates the expression of inflammatory cytokines, including interleukin (IL)-1β, IL-4, IL-6, IL-17α, IL-25, IL-31 and thymic stromal lymphopoietin (TSLP) in mice, and all antioxidants inhibited the upregulation of inflammatory cytokines. ELISA showed the same results as real-time PCR. PM_10_ downregulates the expression of keratinocyte differentiation markers, including loricrin and filaggrin, in mouse keratinocytes and antioxidants prevented the downregulation of the keratinocyte differentiation markers. Conclusively, PM_10_ aggravated the DNCB-induced mouse model in serum IgE concentration, mast cell counts, inflammatory cytokine, and keratinocyte differentiation markers. In addition, antioxidants modulated changes in the DNCB-induced mouse model caused by PM_10_.

## 1. Introduction

Particulate matter (PM) caused by factories, power plants, automobiles, construction activities, and natural windblown dust can be an environmental stressor [1,2]. PM is divided into three subtypes: coarse particles (PM_10_: <10 μm), fine particles (PM_2.5_: <2.5 μm), and ultrafine particles (PM_0.1_: <0.1 μm). These particle sizes differ in origin and health effects. They can damage not only the cardiovascular and respiratory system, but also the skin [3]. They can affect the human body via the synthesis of reactive oxygen species (ROS) [4,5,6,7]. Oxidative stress can cause or aggravate the extrinsic aging of the skin and inflammatory skin disorders [8,9,10,11,12,13].

Antioxidants are used to prevent and/or downregulate oxidative stress and improve inflammatory skin states [14,15,16]. Dieckol is a strong antioxidant in marine algae [17,18,19]. Punicalagin is a natural polyphenol in pomegranate (Punica granatum) and has bioactivities [20,21,22]. Epigallocatechin-3-gallate (EGCG) is the most abundant polyphenol in green tea [16,22]. Resveratrol is a natural product of polyphenolic compounds synthesized by plants in response to infection or other stressors [23,24,25]. Siegesbeckiae Herba extract (SHE) has been used in traditional medicine in many Asian countries [26,27,28]. The effect of phytochemicals and biological function of these compounds have recently been studied [17,18,19,20,21,22,23,24,25,26,27].

In this study, we investigated the effects of antioxidants, including dieckol, punicalagin, EGCG, and resveratrol on PM_10_-induced changes in a 2,4-dinitrochlorobenzene (DNCB)-induced atopic dermatitis (AD) mouse model. In particular, the effects SHE on PM_10_-induced changes in the DNCB-induced atopic dermatitis mouse model were newly investigated.

## 2. Materials and Methods

### 2.1. DNCB-Induced AD Mouse Model

Six-week-old BALB/c mice were purchased from Orient Bio Inc. (Seongnam, Korea) and stabilized for one week. Our animal care and treatment protocols were in accordance with relevant guidelines for Laboratory Animals. Animal experiments were approved by the Institutional Animal Care and Use Committee of the Kyungpook National University (IRB No. KNU 2021-0067).

After one week of stabilization, the back skin of the mice was shaved using clippers and depilated with hair removal cream (6 cm × 6 cm). From the day after hair removal, mice were sensitized with 100 μL of 2% DNCB (2,4-dinitrochlorobenzen) twice a week. DNCB was dissolved in a mixture of acetone and olive oil (3:1). After that, AD-like cutaneous conditions were induced in the mice with 0.2% DNCB applied twice a week for 4 weeks. Mice were treated 100 μL of 100 μg/mL PM_10_ and 100 μL of antioxidants applied to their back skin according to each condition, five times a week for four weeks. Materials were dissolved in PBS. We divided the mice into eight groups (*n* = 3 each); (1) No treatment, (2) DNCB, (3) DNCB + PM_10_, (4) DNCB + PM_10_ + 5 μM dieckol, (5) DNCB + PM_10_ + 5 μM punicalagin, (6) DNCB + PM_10_ + 1 μM EGCG, (7) DNCB + PM_10_ + 1 μM resveratrol, and (8) DNCB + PM_10_ + 10 μg/mL SHE. PM_10_ (PM_10_-like European reference material ERM71 CZ120), dieckol, punicalagin, EGCG, and resveratrol were obtained from Sigma-Aldrich (St. Louis, MO, USA). All the mice were sacrificed by CO_2_ inhalation. Blood, dorsal skin, and spleen samples were obtained and extracted from the mice.

### 2.2. SHE and Its Solvent Fractions

Leaves (140 g) of dried SHE purchased from Sinsun Herb (Seoul, Korea) were ground and extraction was performed at 90 °C for 1 h. The extracted solution was evaporated, and 9 g of the extract was obtained. It was dispersed in 150 mL of water and partitioned sequentially with an equal volume of methylene chloride (MC), ethyl acetate (EA), and n-butyl alcohol (BA). The organic solvents yielded MC fraction (0.27 g), EA fraction (0.16 g), and BA fraction (0.46 g). After filtration, insoluble material (1.15 g) was removed and then evaporated to obtain a water (WT) fraction (6.64 g).

### 2.3. Sebocyte and ORS Keratinocyte Culture

Sebaceous glands were isolated from occipital hairs produced during hair transplantation and were cultivated in Sebocyte Basal Medium (Cell application, San Diego, CA, USA) with Sebocyte Growth Supplement in a 5% CO_2_ incubator at 37 °C. After two weeks of isolation, cells were harvested with 0.25% trypsin/10 mM EDTA in Hank’s balanced salt solution (HBSS) and cultured at EpiLife (Gibco BRL, Rockville, MD, USA).

The sebaceous glands and hair bulb were cut off for the isolation of the outer root sheath (ORS) and were immersed in DMEM supplemented with 20% fetal bovine serum. On the third day of culture, the medium was changed to EpiLife (Gibco BRL) medium containing supplement.

After the second passage, sebocytes and ORS keratinocytes were used for these experiments. Informed written consent was obtained. The Medical Ethical Committee of the Kyungpook National University Hospital approved this study (IRB Number KNUH 2021-03-006-001).

### 2.4. Measurement of Reactive Oxygen Species (ROS)

ROS production in sebocytes and ORS keratinocytes was assessed by measuring 2′,7′-dichlorodihydrofluorescein diacetate (DCF-DA, Invitrogen, Carlsbad, CA, USA). The sebocytes and ORS keratinocytes were seeded onto six-well collagen-coated plates (Becton Dickinson) at 5 × 10^5^ cells/well for 24 h. Pre-labeled cells with 10 μM DCF-DA were washed with PBS for 30 min, and then were treated with 100 μg/mL of PM_10_ and 5 μM punicalagin, 5 μM dieckol, 1 μM EGCG, 1 μM resveratrol, or 10 μg/mL SHE, and incubated at 37 °C for 2 h in the dark. Cells were extracted with 20 mM Tris-Cl buffer containing 1% sodium dodecyl sulfate (SDS) and 2.5 mM ethylene-diamine-tetraacetic acid (EDTA). After centrifuging at 13,000 rpm for 15 min, supernatants were measured using a fluorescence microplate reader (Molecular Devices, Sunnyvale, CA, USA) at excitation at 485 nm and emission at 538 nm.

### 2.5. Measurement of Serum IgE Concentration and Spleen Weight

All mice were sacrificed using CO_2_ and their peripheral blood and spleen were collected. Serum levels of immunoglobulin (IgE) were measured using an enzyme-linked immunosorbent assay (ELISA) kit (Abcam, Cambridge, UK) according to the manufacture’s instructions. Briefly, after adding 100 μL of standard and samples to each well coated with IgE, they were incubated for 30 min at room temperature. After washing four times, they were incubated with 100 μL of an antibody conjugate for 30 min, and then IgE was detected using a microplate reader (Molecular Devices, Sunnyvale, CA, USA) at 450 nm after adding Chromogen Substrate Solution. All spleens were removed immediately after sacrifice and weighed.

### 2.6. Measuring Epidermal Thickness and Counting Mast Cells

The excised dorsal skins of the mice were fixed with 10% formaldehyde and embedded in paraffin. The sections of 6 μm thickness were stained with hematoxylin and eosin (H&E) or toluidine blue (TB). Epidermal thickness was measured in H&E-stained images. Mast cell numbers were counted in TB-stained images. A purple color indicates mast cells.

### 2.7. Real-Time Polymerase Chain Reaction (PCR)

Total RNA was isolated from cutaneous dorsal mouse skin treated with PM_10_ and antioxidants using a TRIzol reagent (Invitrogen, Waltham, MA, USA), and cDNA was synthesized from 3 μg of total RNA using a cDNA synthesis kit containing ImProm-IITM reverse transcriptase and oligo (dT) primers (Promega, Madison, WI, USA) at 72 °C for 10 min and 42 °C for 90 min. PCR primer sequences are summarized in Table 1. Real-time PCR was performed with 50 ng of cDNA and 10 pM primers in the SYBR Green mixture on the Step One Plus Real-Time PCR Assay (Applied Biosystems, Foster City, CA, USA). The cycling conditions for amplification were performed: 95 °C for 10 min, 40 cycles at 95 °C for 15 s, and 60 °C for 60 s. The PCR products were analysed using Step One Plus Real-Time PCR analysis software (Applied Biosystems).

### 2.8. Enzyme-Linked Immunosorbent Assay (ELISA)

To measure the protein levels of interleukin (IL)-1β, IL-4, IL-17α and thymic stromal lymphopoietin (TSLP), skin tissues treated with PM_10_ and antioxidants from dorsal mouse skin were homogenized in lysis buffer (100 mM Tris, 150 mM Nacl, 1 mM EGTA, 1 mM EDTA, 1% Triton X-100, 0.5% SDS and phosphatase/protease inhibitor cocktail) using Tissue Lyser (QIAGEN, Hilden, Germany). The ELISA kits of IL-1β, IL-4, IL-17α, and TSLP (R&D Systems, Minneapolis, MN, USA) were used to measure protein expression. Absorbance was measured at 450 nm using a spectrophotometer (VersaMax Microplate Reader, MA, USA).

### 2.9. Immunofluorescence Staining

The excised skin tissues were embedded in a frozen section compound (Tissue-Tek; Miles, Napierville, IL, USA) in a freezer at −80 °C. Using a cryostat (Leica CM3050 S; Leica, Heidelberg, Germany), they were cut into 8 μm-thick sections and fixed in 4% paraformaldehyde containing 0.1% Triton X-100 for 10 min. Blocking was carried out in 5% donkey serum for 1 h, and incubation with the primary antibody was carried out overnight at 4 °C (loricrin [1:100 dilution; Abcam] or fillaggrin [1:100 dilution; Abcam]). After washing, Alexa Fluor 488-labeled donkey anti-rabbit secondary antibody was used to observe the expression (Molecular Probes, Eugene, OR, USA) and counterstained with 4′6-diamidino-2-phenylindole (DAPI) for 10 min.

### 2.10. Statistical Analysis

Data are expressed as means ± standard deviation (SD). The experiment was performed independently. Analysis of variance (ANOVA) was used for statistical analysis. *p*-values < 0.05 were considered statistically significant.

## 3. Results

### 3.1. An Increase in the Production of Reactive Oxygen Species by PM_10_ in Cultured Sebocytes and ORS Keratinocytes Was Decreased by Antioxidants

**Figure 1:**
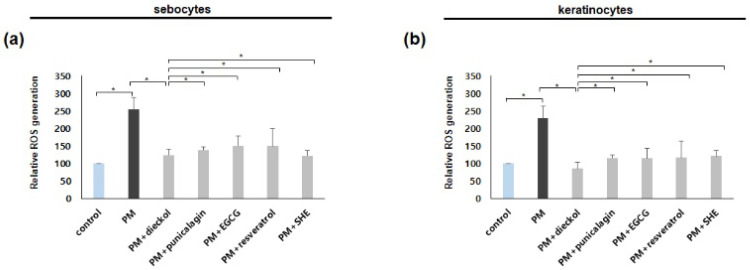
Preventative effects of antioxidants against the upregulation of ROS in the particulate matter <10 µm (PM_10_)-treated sebocytes and ORS keratinocytes. An increase in the production of reactive oxygen species by PM_10_ in cultured (**a**) sebocytes and (**b**) ORS keratinocytes was decreased by dieckol, punicalagin, epigallocatechin-3-gallate (EGCG), resveratrol, and Siegesbeckiae Herba extract (SHE). Data are represented as the mean ± SD from three independent experiments (* *p* < 0.05).

PM_10_ can damage the skin via the synthesis of reactive oxygen species (ROS) [26,29,30]. First, we investigated whether dieckol, punicalagin, EGCG, resveratrol, or SHE inhibited the production of ROS induced by PM_10_. The production of ROS in cultured sebocytes and ORS keratinocytes increased in 100 μg/mL PM_10_. The increased production of ROS by PM_10_ in cultured sebocytes and ORS keratinocytes was inhibited by dieckol, punicalagin, EGCG, resveratrol and SHE (Figure 1).

### 3.2. Preventative Effects of Antioxidants against the Upregulation of Serum IgE Levels and Spleen Weights in the PM_10_-Treated, DNCB-Induced AD Mice

**Figure 2:**
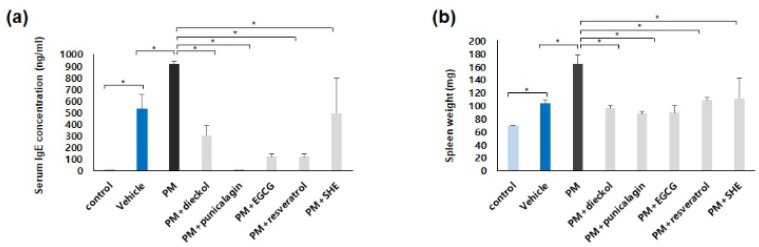
Preventative effects of antioxidants against the upregulation of the serum IgE levels and spleen weights in the particulate matter <10 µm (PM_10_)-treated, 2,4-dinitrochlorobenzene (DNCB)-induced atopic dermatitis mice. (**a**) The serum immunoglobulin E (IgE) levels were significantly decreased after the combined treatment with dieckol, punicalagin, epigallocatechin-3-gallate (EGCG), resveratrol, or Siegesbeckiae Herba extract (SHE) compared with those after PM_10_ treatment only. The data in the bar graphs represent the mean ± standard deviation (SD) from three independent experiments (* *p* < 0.05). (**b**) The spleen weights were also significantly decreased after the combined treatment with dieckol, punicalagin, EGCG, resveratrol, or SHE compared with those after PM_10_ treatment only. The data in the bar graphs represent the mean ± SD from three independent experiments (* *p* < 0.05).

Serum IgE levels were significantly decreased after combined treatment with dieckol, punicalagin, EGCG, resveratrol, or SHE, compared with those after PM_10_ treatment only (Figure 2a). The order of effectiveness was as follows: punicalagin > EGCG = resveratrol > dieckol > SHE. The spleen weights were also significantly decreased after combined treatment with dieckol, punicalagin, EGCG, resveratrol, or SHE, compared with those after PM_10_ treatment only (Figure 2b). The order of effectiveness was as follows: punicalagin = EGCG > dieckol = resveratrol = SHE.

### 3.3. Preventative Effects of Antioxidants against Increased Epidermal Thickness and Mast Cell Counts in the PM_10_-Treated, DNCB-Induced AD Mice

**Figure 3:**
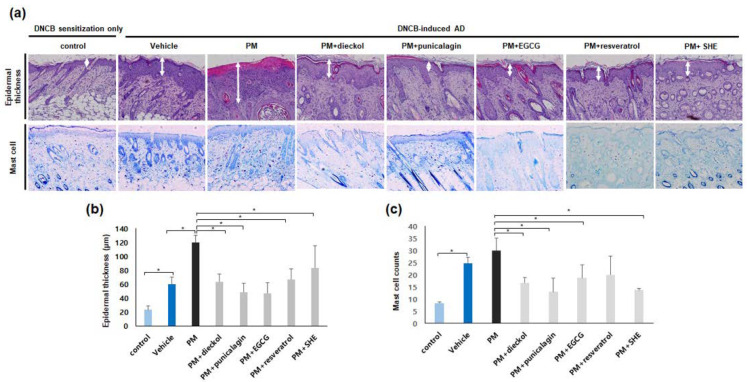
Preventative effects of antioxidants against an increase in epidermal thickness and mast cell counts in the particulate matter <10 µm (PM_10_)-treated, 2,4-dinitrochlorobenzene (DNCB)-induced atopic dermatitis mice. (**a**) Antioxidants showed a decrease in epidermal thickness and mast cell counts in the PM_10_-treated, DNCB-induced atopic dermatitis mice. A white arrows indicate epidermal thickness. The data in the bar graphs represent the mean ± standard deviation (SD) from three independent experiments (* *p* < 0.05). (**b**) Epidermal thickness was significantly decreased after the combined treatment with dieckol, punicalagin, epigallocatechin-3-gallate (EGCG), resveratrol, or Siegesbeckiae Herba extract (SHE) compared with that after PM_10_ treatment only. The data in the bar graphs represent the mean ± SD from three independent experiments (* *p* < 0.05). (**c**) The number of mast cells was also significantly decreased after the combined treatment with dieckol, punicalagin, EGCG, resveratrol, or SHE compared with that after PM_10_ treatment only. The data in the bar graphs represent the mean ± SD from three independent experiments (* *p* < 0.05).

Epidermal thickness was significantly decreased after combined treatment with dieckol, punicalagin, EGCG, resveratrol, or SHE, compared with that after PM_10_ treatment only (Figure 3a,b). The order of effectiveness was punicalagin > EGCG > dieckol > resveratrol > SHE. The number of mast cells was also significantly decreased after combined treatment with dieckol, punicalagin, EGCG, resveratrol, or SHE, compared with that after PM treatment only (Figure 3a,c). The order of effectiveness was punicalagin > SHE > dieckol > EGCG > resveratrol.

### 3.4. Preventative Effects of Antioxidants on the Upregulation of Inflammatory Cytokines in the PM_10_-Treated, DNCB-Induced AD Mice

**Figure 4:**
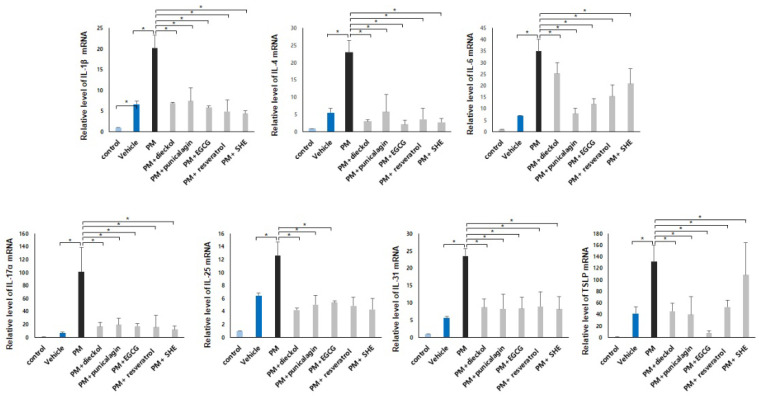
Preventative effects of antioxidants on the upregulation of inflammatory cytokines gene expression in the particulate matter <10 µm (PM_10_)-treated, 2,4-dinitrochlorobenzene (DNCB)-induced atopic dermatitis mice. The gene expressions of interleukin (IL)-1β, IL-4, IL-6, IL-17α, IL-25, IL-31, and thymic stromal lymphopoietin (TSLP) were significantly decreased after combined treatment with dieckol, punicalagin, epigallocatechin-3-gallate (EGCG), resveratrol, or Siegesbeckiae Herba extract (SHE) compared with that after PM_10_ treatment only. The data in the bar graphs represent the mean ± standard deviation (SD) of three independent experiments (* *p* < 0.05).

**Figure 5:**
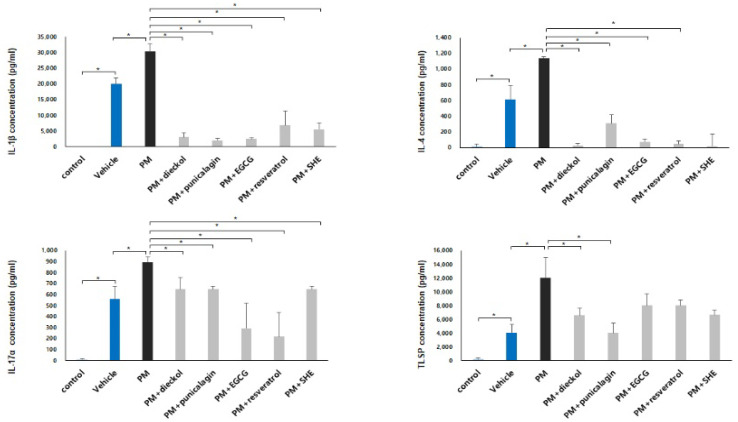
Preventative effects of antioxidants on the upregulation of inflammatory cytokines protein expression in the particulate matter <10 µm (PM_10_)-treated, 2,4-dinitrochlorobenzene (DNCB)-induced atopic dermatitis mice. The protein expression of interleukin (IL)-1β, IL-4, IL-17α, and thymic stromal lymphopoietin (TSLP) was also significantly decreased after combined treatment with dieckol, punicalagin, epigallocatechin-3-gallate (EGCG), resveratrol, or Siegesbeckiae Herba extract (SHE) compared with that after PM_10_ treatment only. The data in the bar graphs represent the mean ± standard deviation (SD) of three independent experiments (* *p* < 0.05).

The gene expressions of IL-1β, IL-4, IL-6, IL-17α, IL-25, IL-31, and TSLP were increased in the DNCB-induced AD mice compared to those in the control group (Figure 4, compare lanes 1 and 2). Surprisingly, when DNCB-induced AD mice were treated with PM_10_, the gene expressions were increased more than in the vehicle-treated group (Figure 4, compare lanes 2 and 3). These results show that PM_10_ can induce atopy by further increasing the expression of genes. Furthermore, the gene expressions of IL-1β, IL-4, IL-6, IL-17α, IL-25, IL-31, and TSLP were significantly decreased after combined treatment with dieckol, punicalagin, EGCG, resveratrol, or SHE, compared to PM_10_ treatment only (Figure 4). The order of effectiveness for the various genes was as follows: IL-1β, SHE > resveratrol > EGCG > dieckol > punicalagin; IL-4 EGCG > dieckol = resveratrol = SHE > punicalagin; IL-6, punicalagin > EGCG > resveratrol > SHE > dieckol; IL-17α, SHE > resveratrol > EGCG > dieckol > punicalagin; IL-25, dieckol = SHE > punicalagin = resveratrol > EGCG; IL-31, SHE > punicalagin > EGCG > dieckol > resveratrol; and TSLP, EGCG > punicalagin > dieckol > resveratrol > SHE. The protein expression of IL-1β, IL-4, IL-17α, and TSLP was also significantly decreased after combined treatment with dieckol, punicalagin, EGCG, resveratrol, or SHE, compared with PM_10_ treatment only (Figure 5). The order of effectiveness for the various proteins was as follows: IL-1β, punicalagin > EGCG > dieckol > SHE > resveratrol; IL-4, dieckol = SHE > resveratrol > EGCG > punicalagin; IL-17α, resveratrol > EGCG > dieckol = punicalagin = SHE; and TSLP, punicalagin > dieckol = SHE > EGCG = resveratrol.

### 3.5. Preventative Effects of Antioxidants on the Upregulation of the Keratinocyte Differentiation Markers in the PM_10_-Treated, DNCB-Induced AD Mice

**Figure 6:**
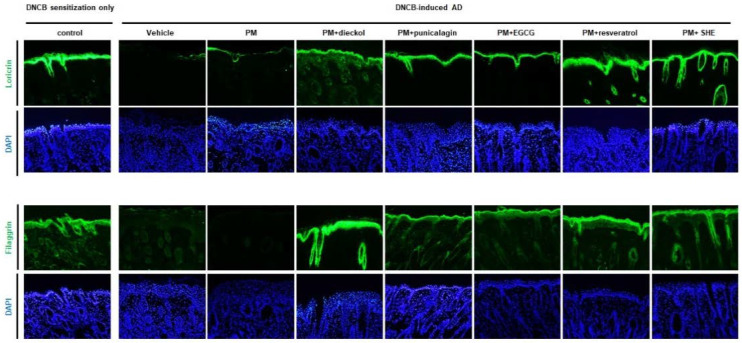
Preventative effects of antioxidants on the upregulation of the keratinocyte differentiation markers in the particulate matter <10 µm (PM_10_)-treated, 2,4-dinitrochlorobenzene (DNCB)-induced atopic dermatitis mice. The downregulation of keratinocyte differentiation markers by PM_10_ alone was prevented by the combination of dieckol, punicalagin, epigallocatechin-3-gallate (EGCG), resveratrol, or Siegesbeckiae Herba extract (SHE).

The downregulation of keratinocyte differentiation markers by PM_10_ was prevented by the combination of dieckol, punicalagin, EGCG, resveratrol, or SHE (Figure 6). The order of effectiveness on loricrin expression was resveratrol = SHE > dieckol = punicalagin = EGCG. The order of effectiveness on filaggrin expression was dieckol > resveratrol = SHE > punicalagin = EGCG.

## 4. Discussion

Air pollution may be related to the development and exacerbation of AD [30,31,32,33]. Woo et al. [34] reported that PM_10_ exposure induces and aggravates skin inflammation via the differential expression of genes controlling skin barrier integrity and immune response in ovalbumin (OVA)-treated mice. Li et al. [35] proved that PM_2.5_ promotes the expression of TSLP in PAM212 cells and may aggravate allergic responses through this pathway. Our study also demonstrated that PM_10_ exposure upregulated the expression of inflammation-related biomarkers and downregulated that of keratinocyte differentiation markers in DNCB-treated mice.

Park et al. [36] established an in vivo model to evaluate PM-induced lung injury in mice. They found that serum IgE levels tended to increase in mice treated with OVA and PM. Cohen et al. [37] showed that there were nominal effects on rat organ weights, including the spleen, as a result of dust exposure at the World Trade Center. Jin et al. [38] suggested that PM may affect mast cell activation in a study using bone-marrow-derived mast cells. Kataoka et al. [39] investigated the cytotoxic effects of water-soluble extracts of coarse and fine atmospheric PM on mast cell lines. Wang et al. reported that PM 2.5 promotes the IgE-mediated mast cell activation of ROS [40]. Several groups [41,42,43] reported that air pollution, including PM_10_, is associated with an increased risk of atopic dermatitis. The study showed that cytotoxic effects and PM concentrations were not always correlated. In our study, PM increased serum IgE levels in DNCB-treated mice. In addition, spleen weights and the number of mast cells were increased in DNCB-treated mice.

The association between PM and AD in humans has been introduced. Park et al. [44] investigated the association between PM and AD and other chronic inflammatory dermatoses using data from the Korean Health Insurance Review and Assessment Service. They concluded that PM was associated with AD and other chronic inflammatory skin diseases. Nakhjirgan et al. [45] also showed a statistically significant relationship between the concentration of PM and the exacerbation of sleep disturbance and itching in Iran. He et al. [46] evaluated the association between PM concentrations and outpatient visits for AD in Lanzhou and concluded that PM was closely related to outpatient visits for AD.

Antioxidants have preventative effects against oxidative stress. Our study revealed that a variety of antioxidants affected serum IgE concentration, spleen weight, mast cell counts, inflammatory cytokines, and keratinocyte differentiation markers in a mouse model of DNCB-induced AD. Punicalagin is more effective than other antioxidants, such as dieckol, EGCG, and resveratrol, for serum IgE concentration, spleen weight, and mast cell counts. The newly studied SHE was also effective in the regulation of serum IgE concentration, spleen weight, mast cell counts, inflammatory cytokines, and keratinocyte differentiation markers in a mouse model of DNCB-induced AD.

Shin et al. [26] reported that resveratrol inhibits PM_10_-induced inflammatory responses in human keratinocytes by reducing aryl hydrocarbon receptor activation and reactive oxygen species formation. Seok et al. [22] also reported that PM_10_-induced inflammatory response is restored by punicalagin and EGCG in keratinocytes. Moreover, Ha et al., [27] reported that dieckol attenuates PM_10_-induced PGE2 production. Recently, we reported that SHE and dieckol inhibit the expression of PM-induced inflammatory cytokines and matrix metalloproteinase in sebocytes and keratinocytes [47,48]. Sargassum horneri is a brown alga with antioxidant, anti-inflammatory, and antiallergic effects. Herath et al. [49] sought to determine whether the ethanol extract of S. horneri mitigated the effect of PM exposure on asthma development. Orally administered S. horneri mitigated the expression of Th2 cytokines in lung homogenates of PM-exacerbated asthmatic mice. In addition, S. horneri markedly mitigated the activation of mast cells and IgE levels in the serum. 2,3,5,4′-tetrahydroxystilbene-2-O-β-d-glucoside (THSG), the main compound of Polygonum multiflorum, has various biological activities, including anti-inflammatory and antioxidant activities. Hwang et al. [50] examined the effects of THSG on Th2 immune responses in an OVA-induced asthma animal model. This study confirmed the potential of THSG in asthma treatment through the modulation of inflammatory responses. Natural functional foods, including antioxidative blueberries and black rice, can be an alternative for AD therapy. Hong et al. [51] investigated the effect of fermented blueberry and black rice extracts containing Lactobacillus plantarum MG4221 on the PM-induced expression of proinflammatory cytokines in vitro and in vivo. Fermented blueberry and black rice extracts decreased IL-1β, IL-6, and IL-8 levels in PM-treated HaCaT cells. In addition, their oral administration significantly decreased transepidermal water loss and the production of serum IgE, and increased the protein expression of filaggrin and involucrin in skin tissue. Polyphenolic compounds, including dieckol, punicalagin, EGCG, and resveratrol, are antioxidative and anti-inflammatory agents. However, the antioxidant activity of SHE has not yet been elucidated.

## 5. Conclusions

In conclusion, PM_10_ upregulated serum IgE concentration and increased spleen weight in the DNCB-induced AD mouse model. In addition, PM_10_ enhanced mast cell counts, inflammatory cytokines, including IL-1β, IL-4, IL-6, IL-17α, IL-25, IL-31, and TSLP, and keratinocyte differentiation markers, including loricrin and filaggrin, in the DNCB-induced AD mouse model. Antioxidants, including dieckol, punicalagin, EGCG, resveratrol, and SHE, had preventative effects against PM_10_ on serum IgE concentration, spleen weight, mast cell counts, inflammatory cytokines, and keratinocyte differentiation markers in the mouse model of DNCB-induced AD. Further studies are needed to investigate the adverse health effects of PM_2.5_ and PM_0.1_ on AD and the preventative effects of antioxidants against PM_2.5_ and PM_0.1_ in AD.

## Figures and Tables

**Table 1:** Real-Time PCR primers used in this study.

Gene	Oligonucleotide Primers	
	Forward	Reverse
mouse GAPDH	AACTTTGGCATTGTGGAAGG	ACACATTGGGGGTAGGAACA
mouse IL-1β	Mm-IL-1β-1-SG (QuantiTect Primer, Qiagen)	
mouse IL-4	ACAGGAGAAGGGACGCCAT	GAAGCCGTACAGACGAGCTCA
mouse IL-6	ACCACTTCACAAGTCGGAGG	TGCAAGTGCATCATCGTTGTTC
mouse IL-17α	ATCCCTCAAAGCTCAGCGTGTC	GGGTCTTCATTGCGGTGGAGAG
mouse IL-25	CGGAGGAGTGGCTGAAGTGGAG	ATGGGTACCTTCCTCGCCATG
mouse IL-31	TCGGTCATCATAGCACATCTGGAG	GCACAGTCCCTTTGGAGTTAAGTC
mouse TSLP	CGGATGGGGCTAACTTACA	TCCTCGATTTGCTCGAACTT

## Data Availability

The data presented in this study are available in the article.
